# Constructive Neutral Evolution 20 Years Later

**DOI:** 10.1007/s00239-021-09996-y

**Published:** 2021-02-19

**Authors:** Sergio A. Muñoz-Gómez, Gaurav Bilolikar, Jeremy G. Wideman, Kerry Geiler-Samerotte

**Affiliations:** School of Life Sciences, Biodesign Center for Mechanisms of Evolution, Arizona State University, Tempe, AZ USA

**Keywords:** Complexity, Adaptation, Neutrality, Entrenchment, Random genetic drift

## Abstract

Evolution has led to a great diversity that ranges from elegant simplicity to ornate complexity. Many complex features are often assumed to be more functional or adaptive than their simpler alternatives. However, in 1999, Arlin Stolzfus published a paper in the Journal of Molecular Evolution that outlined a framework in which complexity can arise through a series of non-adaptive steps. He called this framework Constructive Neutral Evolution (CNE). Despite its two-decade-old roots, many evolutionary biologists still appear to be unaware of this explanatory framework for the origins of complexity. In this perspective piece, we explain the theory of CNE and how it changes the order of events in narratives that describe the evolution of complexity. We also provide an extensive list of cellular features that may have become more complex through CNE. We end by discussing strategies to determine whether complexity arose through neutral or adaptive processes.

## Introduction

Life on Earth exhibits great diversity at multiple levels of organization; for example, among genes in a genome, organelles in a cell, cell types in a multicellular organism, or individuals in a population. This manifold diversity is accentuated by both elegant simplicity and ornate complexity. In general terms, a more complex feature has more part types and/or interactions among parts than a simpler feature (Stoltzfus 1999; McShea and Brandon 2010). For example, the same gene family might comprise one or dozens of members, cells can have a few (prokaryotes) or several organelles (eukaryotes), and organisms can be made of a single cell or trillions. Most complex features are assumed to have more functions or be more adaptive than their simpler alternatives. (Darwin 1859; Dawkins 1996; Lenski 2003) However, this is not always the case. Complex features can evolve either adaptively or non-adaptively, or through a combination of both processes (Gregory 2008; Lynch 2007). In this essay, we discuss an important but under-appreciated theory describing how complex features can evolve in a non-adaptive way without positive selection.

Unnecessarily complex features seemingly abound across the huge diversity of eukaryotes: from extraneous subunits in a protein complex to the convoluted recurrent laryngeal nerve of sauropod dinosaurs and giraffes, which travels from the brain down the long neck to the thorax and then returns to the larynx (Wedel 2011). Perhaps the most straight-forward and best-studied example is that of an ancestral gene whose function, or performance, is subdivided into two paralogs after gene duplication (called subfunctionalization) (Stoltzfus 1999; Force et al. 1999). In this example, the new state is more complex but is not necessarily associated with any immediate adaptive benefit. Some of the most bizarre examples of unnecessarily complex features, however, come from poorly known eukaryotic lineages entirely composed of unicells (protists). For example, ciliates are protists that develop their somatic genome through massive rearrangements from their germline genome. These genomic rearrangements require the massive removal of non-coding DNA, and sometimes even the unscrambling of gene pieces (Braun et al. 2018; Rzeszutek et al. 2020). Another example involves kinetoplastids (which include parasitic protists), that exhibit a dense mass of DNA inside their mitochondria called the kinetoplast. In the kinetoplast, the mitochondrial genome is found distributed in a mess of tangled mini- and maxi-circles whose cryptic genes require massive RNA editing (with the aid of small guide RNAs) to be decoded into functional transcripts and eventually translated into rather unremarkable mitochondrial proteins (Read et al. 2016) What do all these overly complex features have in common and how did they evolve?

These examples portray complex features that are equivalent in function to simpler alternatives. For example, two recent paralogs accomplish the same function as the ancestral gene from which they derive. Both the unscrambling of gene pieces in ciliates or the massive editing of transcripts in kinetoplastids produce proteins functionally equivalent to those of ancestral or sister species lacking these overly complicated processes. A little known but broadly applicable theory has been proposed to explain how this complexity can arise via neutral processes. The theory was born out of a group of molecular evolutionists at Dalhousie University in the 1990s. The first sketches of this theory were published by Covello and Gray (1993), who proposed a neutral theory for the evolution of RNA editing (Covello and Gray 1993). They drew inspiration from the Osawa and Jukes 1989 model for the neutral evolution of alternative genetic codes across animal mitochondria (Osawa and Jukes 1989). This theory was then generalized and given its name, “Constructive Neutral Evolution” (CNE), by Arlin Stoltzfus in a seminal paper published in the Journal of Molecular Evolution in 1999. In this paper, Stolzfus describes how the origin, maintenance, or further complexification of features like the scrambled gene pieces of ciliates, mRNA pan-editing in kinetoplastid mitochondria, the spliceosome, and gene paralogs can occur through neutral processes (Stoltzfus 1999). Others have since further popularized CNE and used it to explain an even broader range of complex features (Gray et al. 2010; Lukeš et al. 2011; Brunet and Doolittle 2018). However, many evolutionary biologists still appear to be unaware of this explanatory framework for the origins of complexity.

There might be a few reasons why CNE has not been more widely embraced. First, some of the most iconic examples of CNE are from lesser studied single-celled protists. These examples might be considered obscure or idiosyncratic of only a few wildly divergent lineages by biologists more familiar with model organisms and multicellular eukaryotes. Second, most CNE examples appear to be restricted to genes or multi-protein complexes and, therefore, the generality and applicability of CNE to other levels of organization (e.g., tissues, populations, ecological communities, and ecosystems) is less clear. Lastly, complex features such as macromolecular machines are typically thought of as having evolved gradually and been shaped to near-perfection via adaptation and natural selection; this thinking echoes Darwin’s original ideas about the evolution of complex organs such as the eye (Darwin 1859).

## What Is Constructive Neutral Evolution (CNE)?

CNE describes a process by which complexity can arise or increase in a neutral fashion. This means that increases in complexity are not necessarily advantageous and positive selection is not required for their evolution.

The word ‘constructive’ in CNE refers to an increase in complexity, in contrast to ‘reductive’ or ‘conservative’ (Stoltzfus 1999). The word ‘neutral’ in CNE does not necessarily refer to the vernacular meaning of the word, where a feature is neutral when it does not provide a fitness advantage or disadvantage to its possessors. Instead, the word ‘neutral’ refers to the concept of ‘effective neutrality’ from population genetics (Kimura 1968, 1979; Ohta 1973), which calls effectively neutral any feature, whether advantageous or disadvantageous, that spreads out in a population by random genetic drift. For example, a gene duplication event can be disadvantageous to fitness because it changes gene dosage (Papp et al. 2003). However, if this disadvantage is small enough, the duplication might escape natural selection and passively drift to a higher frequency. This is particularly likely in populations with small effective population sizes where a stochastic process like genetic drift can overwhelm a deterministic process like natural selection.

CNE, as formulated by Stolzfus, is a process that relies on the following five concepts: (1) excess capacities, (2) epistasis, (3) random genetic drift, (4) biased variation, and (5) purifying selection. Even though random genetic drift and purifying selection are not explicitly discussed by Stoltzfus (1999), these are population-level processes required for evolutionary change and persistence. We dissect each one of these concepts below, provide examples, and explain how they each contribute to CNE.

*Excess capacities* are components or properties of a system (e.g., a cell or organism) that have not been selected for, or have non-selected side effects, and whose removal would not be harmful (Stoltzfus 1999). Excess capacities have thus also been called ‘gratuitous’ or ‘unsolicited’ (Brunet and Doolittle 2018; Lukeš et al. 2011; Stoltzfus 1999). For example, a new gene duplicate immediately creates an excess capacity, which as a consequence, relaxes functional constraints on each gene copy (Force et al. 1999; Stoltzfus 1999; Ohno 1970). Many other examples of excess capacities refer to features that are, at least to some extent, substrate-indifferent, which means that they can interact with a large range of similar substrates. For example, tRNA–aminoacyl synthetases specifically bind their cognate tRNAs, but they can also occasionally bind to unrelated RNAs that somewhat resemble tRNAs in their tertiary structure (Tworowski et al. 2005; Bullwinkle and Ibba 2014). Another example is the hypothesized ‘editisome’ precursor in kinetoplastid mitochondria (Covello and Gray 1993). This prototypal RNA-editing machinery is hypothesized to have been composed of enzymes that carried out functions other than those required for RNA editing in the kinetoplastid. However, when this set of enzymes associated with the first gRNAs, the emergent machinery could not only edit one site in one transcript but dozens of sites in multiple transcripts. Excess capacities are, therefore, a prerequisite for CNE, and their role is to allow for previously forbidden mutations to occur, i.e., they allow for a specific kind of epistasis.

*Epistasis* occurs when the phenotypic effects of a mutation are context dependent (Eguchi et al. 2019), specifically when they depend on other mutations or genes. In CNE, epistasis is a consequence of excess capacities. For example, the presence of a gene duplicate suppresses, or renders neutral, loss-of-function mutations in the other gene duplicate. These conditionally neutral mutations would have been deleterious in the absence of a gene duplicate (i.e., the excess capacity). In the case of kinetoplast mRNA pan-editing, the editisome allows for deletions or insertions (‘indels’) to happen in the target ‘cryptogene’ without any harmful phenotypic consequences or fitness decline. Epistasis, thus, masks the harmful effects of mutations, and releases selective constraints. In the absence of all or most selective constraints, some changes can accumulate as a consequence of random genetic drift.

*Random genetic drift* allows for neutral, or even slightly deleterious, mutations to increase in frequency or become fixed in populations; this becomes much more probable in species with small effective population sizes. For example, mutations that decrease function in a recently duplicated gene can drift to fixation because of the redundancy provided by an extra gene copy (Force et al. 1999; Stoltzfus 1999). Similarly, a large number of indels in cryptogenes are made neutral by the excess capacity provided by the editisome, and might therefore spread in the population by drift. The role of random genetic drift in CNE is to allow for the chance fixation of conditionally neutral mutations whose potentially harmful effects have been suppressed by an excess capacity. Because excess capacities largely diminish selective constraints, the mutations that are fixed via genetic drift may reflect inherent mutational biases.

*Biased variation* occurs when certain mutational changes are more common than others. Some biases tend to lead to disordered or more complex states and are inherent to a system (‘systemic’ or ‘global’ biases; Stoltzfus 1999, 2012). For example, when an ancestral gene has just duplicated, mutations that reduce function are more common than those that confer new functions (Stoltzfus 1999; Force et al. 1999; Levasseur and Pontarotti 2011; Proulx 2012). Similarly, deletions in kinetoplast cryptogenes are much more common than insertions. Perhaps the clearest example comes from ciliates which have their genes scrambled out of order in their germline genomes. Once a mechanism to unscramble gene pieces evolves, these pieces will inevitably get more re-arranged and scattered because mutations that scramble pieces are common, while mutations that put pieces back into exactly the right spot are rare. Biased variants that have been fixed by random genetic drift create or enhance the (inter-)dependencies between excess capacities and their substrates. In this way, effectively neutral biased variation provides directionality to CNE. Once (inter-)dependencies evolve, they are maintained by purifying selection.

*Purifying selection* purges deleterious mutations from populations. One key role of purifying selection during CNE is to prevent dissociation of neutrally evolved (inter-)dependencies. For example, once the first indels have occurred in the incipient cryptogenes, purifying selection prevents any mutations that might disrupt the function of the editisome. Likewise, once gene paralog pairs have become essential through reciprocal loss-of-function mutations, purifying selection prevents the loss of either paralog. In addition to preventing the loss of complexity that has been created via CNE, purifying selection also results in the phenotypic effects (‘higher-level’ or ‘downstream’ phenotype) of the complex features in question remaining relatively unaltered and prevents fitness from declining considerably (Wideman et al. 2019); (Zhang 2018). For example, purifying selection prevents mutations other than indels from accumulating in cryptogenes, or further loss-of-function mutations in gene paralogs that have already become essential through reciprocal loss-of-function mutations. In this way, purifying selection acts as a sieve that purges deleterious mutations but allows the accumulation of mutations whose phenotypic effects are buffered (Geiler-Samerotte et al. 2016, 2019).

**Fig. 1:**
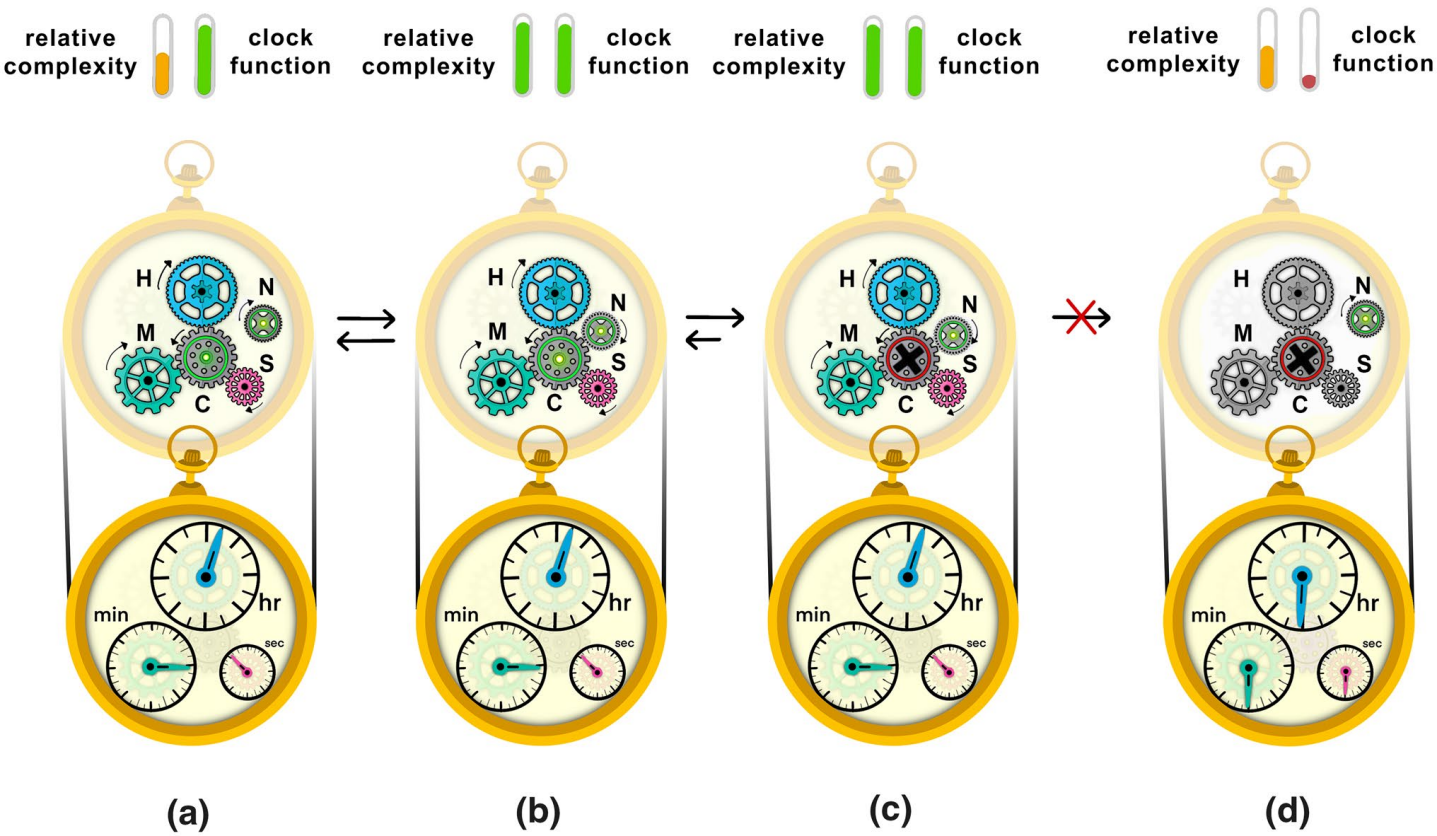
Ratchet-like processes build-up complexity by increasing the number of gears that underlie clock function. In a hypothetical clock, three gears (Gear H, Gear M, and Gear S) control the dials (hr, min, and sec, respectively). They are put in motion by the central gear (Gear C), which is powered by the battery (indicated by the green light at its center). From panel **a** to **b** the clock increases in complexity. Step **a** to **b** indicates the addition of a previously non-interacting gear (Gear N), which does not affect the function of the clock. Step **b** to **c** introduces a defect in the central gear (Gear C), making the new gear (Gear N), which has its own power source, essential for the clock to function. Step **c** to **d** separates the new gear (Gear N) from the rest of the machinery. This change renders the clock non-functional due to lack of access to power. In sum, the presence of a new powered gear (Gear N) introduces excess capacity, which provides the opportunity for another gear (Gear C) to lose power and results in an increase in complexity that is difficult to revert

To summarize, the excess capacities of a biological system first allow for gratuitous interactions to occur between two or more components (Fig. 1). These interactions result in a particular type of epistasis in which a previously harmful mutation is made effectively neutral. Once a previously harmful mutation has been made less harmful or neutral, it might drift to fixation. If it does, this fixed mutation ‘locks in’ the previously gratuitous interaction. In other words, an (inter-)dependency has now emerged, and as a direct result, complexity has also increased (i.e., the number of interacting parts has grown). This has happened in a neutral fashion; in other words, the changes have been established by random genetic drift, even if slight decreases in fitness have occurred. There is no positive selection at any step in this build-up of complexity. Because the newly evolved (inter-)dependency is now essential, purifying selection prevents the dissociation of the more complex, neutrally evolved feature. Dissociation of the (inter-)dependency would disrupt function and decrease fitness by exposing the conditionally harmful mutation (Fig. 1).

## Alternative Frameworks for Thinking About CNE

While Stoltzfus talks about excess capacity, epistasis, and biased variation, Gray and colleagues use a different language when discussing neutral processes that increase complexity. These authors have described CNE as a neutral ratchet-like process that results in ‘irremediable complexity’ (Gray et al. 2010). The complex features that CNE produces have been described as the consequence of ‘runaway bureaucracy’ or ‘biological Rube Goldberg machines’ which are unnecessarily complex and over-engineered to perform a single task (Lukeš et al. 2011). It has also been said that CNE allows for ‘function diffusion’ in the sense that the same function now depends on more part types or interactions (Lukeš et al. 2011), or that CNE allows for a structure to ‘degenerate its way into complexity’ (Zimmer 2013).

The term ‘pre-suppression’ takes a central role in the conceptual framework developed by Gray and colleagues. Pre-suppression happens when otherwise harmful or prohibited changes are made possible or neutral because of the presence of gratuitousness in a biological system. This concept thus refers to the epistatic effects that excess capacities confer. Subsequently, once a previously prohibited mutation occurs, its otherwise harmful consequences are effectively ‘suppressed’. This stage corresponds to the establishment of a new dependency; a complete turn of the CNE ratchet. Pre-suppression often allows for multiple neutral changes to accumulate. This can increase the dependency between two or more components. For example, a protein ‘A’ (client) that depends on protein ‘B’ (chaperone) for stability or proper folding might accumulate several destabilizing mutations that increase its dependency on protein ‘B’. The key point is that an (inter-)dependency, and the increase in complexity that it represents, becomes more difficult to reverse as more neutral changes accumulate. This effective irreversibility is why CNE is described as a ratchet-like process (Gray et al. 2010; Lukeš et al. 2011).

## Comparing CNE to Other Evolutionary Narratives

**Fig. 2:**
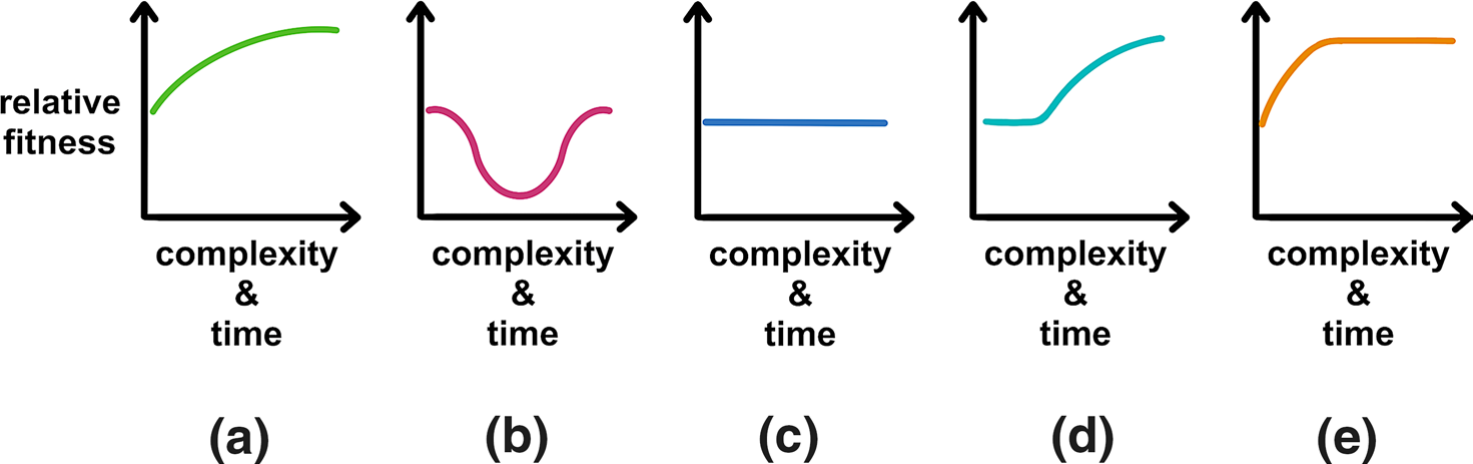
Evolutionary narratives that describe the evolution of complex features differ in the trajectories that relative fitness follows as well as in the number of steps required. **a** A traditional evolutionary tale for the progressive evolution of complex adaptations. **b** ‘Compensatory’, ‘rescue’, ‘error-correcting’ or ‘post-suppressive’ narrative for the evolution of certain complex features. **c** CNE narrative (also known as ‘pre-suppressive’ narrative). **d** Exaptive narrative where neutral evolution precedes adaptive evolution. **e** Combination of an adaptive phase that is followed by neutral evolution. All evolutionary narratives portrayed here are assumed to increase complexity

Traditional narratives that explain the evolution of complex features assume that complexity is adaptive, i.e., a more complex feature must endow its possessor with a competitive advantage. This view also often proposes that complexity evolves in a gradual manner where each gain in complexity is favored relative to its simpler precursors (Fig. 2a) (Zimmer 2013). Darwinian positive selection is thus the putative driving force throughout the whole process.

Another narrative that invokes positive selection to explain the origin of complexity involves two stages. In the first stage, one or several mutations result in a fitness disadvantage which creates a selection pressure for a compensatory mutation to restore fitness (Fig. 2b). This can occur in several ways. For example, an increased number of deleterious mutations might accumulate because of a transient environmental change that temporarily suppresses their harmful effects (e.g., transient hypoxia suppresses mitochondrial mutations) (Cavalier-Smith 1993). Alternatively, deleterious mutations could hitchhike if they are linked to an advantageous mutation (e.g., mutator alleles in laboratory-evolved *E. coli* populations) (Sniegowski et al. 1997). In either case, the deleterious mutation creates a selection pressure that leads to the second stage of the narrative: the evolution of an ‘error–correction’ mechanism that is inherently more complex than reversing the harmful mutation (Cavalier-Smith 2005). This narrative has been used to explain the origin of pan-editing in kinetoplastid mitochondria (Cavalier-Smith 1993). This type of narrative implies a (sometimes drastic) decline in fitness that is subsequently restored by an evolutionary novelty which is driven by positive selection because it ‘post-suppresses’ the harmful mutations (Fig. 2b). A somewhat similar narrative involves mildly deleterious, though effectively neutral, mutations rising in frequency through genetic drift. These interact with subsequent mutations to create adaptive increases in complexity that were not previously possible, and that rise in frequency through positive selection (ZuckerkandI 1997).

In contrast to these previous narratives, the order of events is reversed in CNE narratives. It is often claimed that ‘post-suppressive’ or ‘error-correcting’ narratives put the cart before the horse (Gray et al. 2010). In a CNE narrative, a ‘pre-suppressive’ excess capacity is available beforehand. This property buffers the harmful consequences of a conditionally deleterious mutation, thus allowing it to become neutral and then spread in the population through random genetic drift. Therefore, there is no mal-adaptive stage where the organism faces a serious competitive disadvantage relative to its sisters and ancestors; nor is there a phase where positive selection is involved (Fig. 2c). Instead, the whole process happens in an effectively neutral fashion and there are no fitness gains. Pre-suppressive CNE narratives are thus well-suited to explain the evolution of many complex features, especially when the increase in complexity does not affect higher-level phenotypes (Wideman et al. 2019).

These different evolutionary narratives are not mutually exclusive and can work in concert to produce complex features. For example, complex structures built neutrally can provide a ‘substrate’ for subsequent adaptation to create new functions or hone pre-existing ones; this represents an example of exaptation (Fig. 2d). For example, it seems likely that the intricate editisome of kinetoplastid mitochondria has been further shaped by selection to improve the efficacy of transcript editing, and subsequently perhaps, this has allowed for more gene encryption. It is also conceivable that the first ribosomes were pure ribozymes whose occasional structural defects were pre-suppressed by ‘stabilizing’ proteins. The neutral accretion of proteins to a ribozyme core provided a more flexible ‘substrate’ for natural selection to build a much more efficient ribosome through exaptation (Lukeš et al. 2011; Noller et al. 1992; Ban et al. 2000; Fox 2010).

In an opposite way, certain adaptations release constraints on organismal parts or structures which can then accrue complexity non-adaptively. In other words, adaptation can produce excess capacities which in turn can lead to CNE (Fig. 2e). It is thought, for example, that the origin of the nuclear envelope enabled the spread of gene-interrupting introns in eukaryotic genomes as a side effect (Cavalier-Smith 1993). One can also imagine that the origin of the macronucleus in ciliates (an adaptation that increased gene dosage to support giant active cells) allowed for transposon-like DNA elements to spread in a silenced germline micronucleus (Cavalier-Smith et al. 2004).

## A Wide Diversity of Complex Features Can Be Explained By CNE

Most of the features plausibly explained by CNE refer to either genes or macromolecular machines. In order to make his case for the possibility of CNE, Stoltzfus presented four detailed examples: the subfunctionalization of gene duplicates, the origin and complexification of the spliceosome, the origin and increase of RNA pan-editing in kinetoplastid mitochondria, and the scrambling of gene pieces in the germline nuclei of ciliates (Stoltzfus 1999). Each of these examples is supported by logical arguments, observations, and also by different degrees of experimental and comparative evidence. The case for the origin and evolution of the spliceosome is best supported by experimental and comparative evidence: proteins structurally compensate for degenerated RNA structures and the ancestors of spliceosomal introns were self-splicing (Stoltzfus 1999). The case for gene scrambling and RNA pan-editing relies more on logical arguments rather than empirical evidence: once the unscrambling and pan-editing machinery are in place, scrambled genes and edited sites are expected to increase in frequency without a significant cost (Covello and Gray 1993; Stoltzfus 1999). Finally, the subfunctionalization of gene duplicates is supported by a model whose predictions match observations on the tempo and mode of evolution of recent paralogues (Stoltzfus 1999; Force et al. 1999).

Since Stolzfus’s seminal paper in the Journal of Molecular Evolution, CNE has been applied to describe the evolution of many other complex cellular features including the ribosome, the replisome, the proliferation of transposons in eukaryotic genomes, the interdependence between endosymbiotic organelles and their host cells, the metabolic division of labor in insect nutritional symbionts, mitochondrial respiratory complexes, light-harvesting antennae in algae, protein folding and import machinery, the cytoskeleton and its associated motors, gene-regulatory network architecture, and trans-splicing in diverse eukaryotes (e.g., roundworms, kinetoplastids, and dinoflagellates) (Van Leuven et al. 2014; Gray et al. 2010; Britton et al. 2020; Sorrells and Johnson 2015; Roger et al. 2017; Lukeš et al. 2009). The evidence for most of these examples appears to rely primarily on comparative data and functional reasoning. Most of these complex features exist in simpler forms in some lineages without incurring any apparent change in performance, stability, or efficiency. Nevertheless, most, if not all of these proposed examples remain speculative and await further evidence and detailed evolutionary narratives. However, a few putative examples of CNE have been the subject of more detailed experiments (Finnigan et al. 2012; Britton et al. 2020; Hochberg et al. 2020). Table 1 lists some examples of complex features and the components required to explain how neutral evolutionary processes contributed to their complexity.

**Table 1:** Some complex features whose evolution is potentially explained by CNE

Complex feature	Phylogenetic breadth (age)	Excess capacity (realizes suppressive epistasis)	Biased variation (fixed through random genetic drift)	(Inter-) dependency	References for CNE view	References for alternative selective view
Gene duplication	All taxa	Redundant second copy of a gene	Loss-of-function mutations are more probable than gain-of-function mutations	Reciprocal loss-of-function mutations render each duplicate essential to maintain function	Lynch et al. (1999) and Stoltzfus (1999)	Kondrashov and Kondrashov (2006) and Kondrashov and Koonin (2004)
Scrambled genes	Ciliates	Sites that flank transposon-like insertions and unscrambling machinery that recognizes those sites	Further scrambling of gene pieces is more probable than mutational unscrambling	Proliferation of transposon-like insertions make the unscrambling machinery essential	Katz and Kovner (2010), Prescott and Greslin (1992) and Stoltzfus (1999)	Katz and Kovner (2010) and Maurer-Alcalá and Nowacki (2019)
gRNA-mediated pan-editing	Kinetoplastids	gRNA and pre-existing precursor editisome	Small deletions are more probable than small insertions	Accumulations of deletions in primordial cryptogenes make the precursor editisome essential	Covello and Gray (1993) and Stoltzfus (1999)	Speijer (2006), Landweber and Gilbert (1993) and Stuart (1993)
Spliceosome	Eukaryotes	Modular function of group II intron ribozymes, trans-acting snRNAs, and RNA-stabilizing proteins	RNA structure-disrupting or loss-of-function mutations are more probable	Structural defects in group II introns make trans-acting snRNAs and proteins essential	Stoltzfus (1999)	Bandea (2009) and Nilsen (2003)
Spliceosomal intron spread in eukaryotic genomes	Eukaryotes	A nuclear envelope that de-coupled transcription from translation, and the spliceosome	Further spliceosomal intron insertion into genes is more probable than their precise mutational removal	Proliferation of spliceosomal introns in nuclear genomes makes the spliceosome essential for their splicing from pre-mRNAs	Cavalier-Smith (1991)	Bandea (2009)

Note that excess capacities allow for effectively neutral biased variation to accumulate through random genetic drift, thereby creating (inter-)dependencies

The majority of complex features that typically fall under the scope of CNE are restricted to genetic loci, protein–RNA, and protein–protein interactions. However, CNE theory might also help explain the evolution of gene regulatory networks, in particular how their rate-limiting steps change under stabilizing selection (Orlenko et al. 2016a,b), as do their topologies and level of connectedness (Britton et al. 2020; Sorrells and Johnson 2015). Indeed, the scope of CNE has recently been extended to higher levels of biological organization, including the topology and degree of modularity in protein–protein interaction networks, the complexification of the eukaryotic cells, and the division of labor in microbial communities (Brunet and Doolittle 2018). This hierarchical view of CNE also appears to place more emphasis on how (inter-)dependencies increase between parts, rather than on the accretion of new part types (Brunet and Doolittle 2018).

There are some fairly generic genomic features of eukaryotic cells that may have originated or increased in complexity through CNE. For example, similar to the sub-functionalization of paralogue gene pairs, successive sub-functionalization can also explain the expansion of numerous gene families in eukaryotes. Additionally, the expansion of non-coding DNA in most eukaryotic genomes may have been fueled by excess capacities. For example, the proliferation and expansion of introns was possible only once a high-throughput spliceosome had evolved. And the invasion and spread of transposable elements might have followed the origin of efficient machinery for gene silencing. A similar argument can be applied to organelle genomes which now harbor great numbers of defunct (fossilized and ORF-less) group II introns, as long as a few introns and their respective maturases remain functional (Muñoz-Gómez et al. 2017; Hallick et al. 1993). These examples show that CNE might have played an important role in genome evolution across eukaryotes.

## How Do We Identify Features that Evolved Through CNE?

In a most general sense, CNE features are those that are more complex and yet do not improve fitness relative to their simpler ancestral homologues. Even though we cannot directly study ancestral phenotypes, we can compare a candidate CNE feature to its sister homologues using a phylogenetic framework. This approach allows us to infer whether a feature is more complex than its inferred ancestor. The adaptiveness of a feature is, on the other hand, much more difficult to infer. One method for doing so, without direct experimentation, uses a comparative approach and makes inferences about the immediate phenotypic impacts of the feature in question. If the downstream phenotypic effects of a complex feature are the same as that of simpler versions of the feature found in sister lineages, the complexification might not have added any new function. Complexity might therefore be considered to have arisen through CNE processes (Zhang 2018; Wideman et al. 2019). For example, consider a massively edited RNA transcript and an unedited RNA transcript that both produce the same functional protein after translation. Or consider the synthesis of a small set of respiratory complex core subunits in mitochondria (encoded by either seven or twelve protein-coding genes in the mitogenomes of yeast or trypanosomes, respectively) that is equally achieved in a 73-subunit mitoribosome (in yeast) (Desai et al. 2017) and in a protein-rich 126-subunit mitoribosome (in trypanosomes) (Ramrath et al. 2018). Both of these examples showcase needless complexities that, most probably, do not add any new functions or fitness benefits.

The above approach is indirect as it relies on inferences and does not involve direct experimentation. It has been used to suggest which complex features potentially evolved through CNE (Lukeš et al. 2009, 2011; Gray et al. 2010). Such an approach is more easily applied to complex features that evolved more recently and are therefore phylogenetically restricted to fewer taxa. This is because derived features can be more easily compared to their sisters and inferred ancestors (i.e., the degree of divergence is lower), and have less complicated histories (i.e., their evolution is less confounded by subsequent secondary adaptation or exaptation). Functional inferences are therefore made in a more straightforward manner. Notable examples of phylogenetically restricted CNE features are (1) the requirement of certain introns on a tyrosyl–tRNA synthetase for splicing in the mitochondrial genome of the fungus *Neurospora* (Akins and Lambowitz 1987), (2) the editing of a single position in the aspartyl–tRNA of marsupial mitochondria (BÖrner and Pääbo 1996), and (3) several chaperonin paralogues in relatively closely related archaeal species (Archibald et al. 1999).

The same indirect approach can also be applied to more ancient features. The spliceosome and ribosome are, for example, ancient ribonucleoprotein complexes whose evolution might also have been shaped by CNE. Complex features with long evolutionary histories, however, are unlikely to be purely the outcome of a single evolutionary process. CNE might only explain certain aspects of ancient complex features such as their origin, elaboration (i.e., complexification), or maintenance (e.g., constraints that prevent loss). However, we can assume that because most ancient features play central roles in the intricate biology of modern organisms, these primarily evolve under stabilizing selection for a conserved function (Lynch and Trickovic 2020; Lynch 2018). Since stabilizing selection only permits mutations that do not alter phenotype, much of the divergence seen in ancient complex features across the tree of life could be effectively neutral. An implication of this idea is that most divergence will be structural rather than functional, e.g., in subunit number or size (Muñoz-Gómez et al. 2020; Lynch 2013). This structural divergence might be more pronounced in the macromolecular machines of lineages with smaller effective population sizes (like many animals and plants) where the force of random genetic drift is stronger. Indeed, macromolecular machines in prokaryotes, which often have the largest effective population sizes, are often streamlined (e.g., ribosomes and respiratory complexes; Elurbe and Huynen 2016; Petrov et al. 2019; Melnikov et al. 2018). Comparative structural and functional analysis of ancient molecular macro-machines across closely and distantly related species may provide additional examples of candidate features that gained complexity via CNE.

Some studies take a more direct approach to make inferences about the evolutionary history of complex features. In such studies, ancestral versions of the feature (Finnigan et al. 2012; Pillai et al. 2020; Hochberg et al. 2020), or intermediate versions on the path to increased complexity (Britton et al. 2020), are reconstructed and their functional properties assayed. In our opinion, this more direct approach constitutes the ‘gold standard’ for testing CNE narratives. For example, this approach was used to express an ancestral version of a vacuolar V-ATPase proton pump in modern yeast cells in order to assess its function (Finnigan et al. 2012). Unlike in most eukaryotes, the membrane-embedded rotor (c-ring of the V_O_ domain) of the fungal V-ATPase is a decamer composed of three instead of two paralogous subunits. The extra subunit arose when a subunit of the ancestral two-paralogue c9c’ decameric c-ring gave rise to a new paralogue yielding a c8c’c’’ ring. The c’’ subunit was made essential by the reciprocal loss of complementary binding interfaces. This did not confer any novel functions to the V-ATPase, and so it could plausibly represent a neutral increase in complexity (Finnigan et al. 2012). In contrast, a reconstruction of the evolutionary history of vertebrate tetrameric hemoglobin shows that while the initial dimerization may have been neutral, the next step, tetramerization, immediately provided new beneficial functional properties such as cooperativity (Pillai et al. 2020). These kinds of studies show that it is possible to improve inferences about whether a feature became more complex via adaptive or neutral processes by using modern phylogenetic and experimental methods.

## Open Questions

Although there are some direct criticisms of CNE in the literature (Speijer 2006), there are several other conceptual matters surrounding CNE that remain to be explored. The concept of excess capacities or 'pre-suppression', for example, plays a fundamental role in CNE theory. However, several questions about excess capacities come to mind: are they themselves costly? Does their suppression capacity have a limit? CNE also remains a largely verbal and purely qualitative theory. Future attempts at developing a quantitative mathematical theory for CNE will improve its predictability and testability.

## Conclusion

The theory of Constructive Neutral Evolution has been around for more than two decades. Despite this, few molecular and evolutionary biologists seem to be familiar with it. However, as we have shown here, the explanatory scope of CNE is potentially very large. We hope that our perspective provides some food for thought about the processes that sculpt important biological features, specifically the processes that cause them to diversify, expand, and become more complex. Our view is that by considering neutral processes like CNE, in addition to adaptive processes, richer and more accurate evolutionary narratives might be constructed for the origins of complex features.

## References

[CR1] Akins RA, Lambowitz AM (1987). A protein required for splicing group I introns in neurospora mitochondria is mitochondrial tyrosyl-tRNA synthetase or a derivative thereof. Cell.

[CR2] Archibald JM, Logsdon JM, Doolittle WF (1999). Recurrent paralogy in the evolution of archaeal chaperonins. Curr Biol.

[CR3] Ban N, Nissen P, Hansen J (2000). The complete atomic structure of the large ribosomal subunit at 2.4 A resolution. Science.

[CR4] Bandea C (2009). A unifying scenario on the origin and evolution of cellular and viral domains. Nat Preced.

[CR5] BÖrnerPääbo GVS (1996). Evolutionary fixation of RNA editing. Nature.

[CR6] Braun J, Nabergall L, Neme R (2018). Russian doll genes and complex chromosome rearrangements in *Oxytricha trifallax*. Genes Genomes Genet.

[CR7] Britton CS, Sorrells TR, Johnson AD (2020). Protein-coding changes preceded cis-regulatory gains in a newly evolved transcription circuit. Science.

[CR8] Brunet TDP, Doolittle WF (2018). The generality of constructive neutral evolution. Biol Philos.

[CR9] Bullwinkle TJ, Ibba M (2014). Emergence and Evolution. Top Curr Chem.

[CR10] Cavalier-Smith T (1993). The eukaryotic genome: organization and regulation.

[CR11] Cavalier-Smith T (1991). Intron phylogeny: a new hypothesis. Trends Genet.

[CR12] Cavalier-Smith T (2005). Economy, speed and size matter: evolutionary forces driving nuclear genome miniaturization and expansion. Ann Bot.

[CR13] Cavalier-Smith T, Hirt R, Homer D (2004). Chromalveolate diversity and cell megaevolution: interplay of membranes, genomes and cytoskeleton. Organelles, genomes and eukaryote phylogeny.

[CR14] Covello PS, Gray MW (1993). On the evolution of RNA editing. Trends Genet.

[CR15] Darwin C (1859). On the origin of species.

[CR16] Dawkins R (1996). The blind watchmaker: why the evidence of evolution reveals a universe without design.

[CR17] Desai N, Brown A, Amunts A, Ramakrishnan V (2017). The structure of the yeast mitochondrial ribosome. Science.

[CR18] Eguchi Y, Bilolikar G, Geiler-Samerotte K (2019). Why and how to study genetic changes with context-dependent effects. Curr Opin Genet Dev.

[CR19] Elurbe DM, Huynen MA (2016). The origin of the supernumerary subunits and assembly factors of complex I: a treasure trove of pathway evolution. Biochim Biophys Acta.

[CR20] Finnigan GC, Hanson-Smith V, Stevens TH, Thornton JW (2012). Evolution of increased complexity in a molecular machine. Nature.

[CR21] Force A, Lynch M, Pickett FB (1999). Preservation of duplicate genes by complementary, degenerative mutations. Genetics.

[CR22] Fox GE (2010). Origin and evolution of the ribosome. Cold Spring Harb Perspect Biol.

[CR23] Geiler-Samerotte K, Sartori FMO, Siegal ML (2019). Decanalizing thinking on genetic canalization. Semin Cell Dev Biol.

[CR24] Geiler-Samerotte KA, Zhu YO, Goulet BE (2016). Selection transforms the landscape of genetic variation interacting with Hsp90. PLOS Biol.

[CR25] Gray MW, Lukeš J, Archibald JM (2010). Irremediable complexity?. Science.

[CR26] Gregory TR (2008). The evolution of complex organs. Evol Educ Outreach.

[CR27] Hallick RB, Hong L, Drager RG (1993). Complete sequence of *Euglena gracilis *chloroplast DNA. Nucleic Acids Res.

[CR28] Hochberg GKA, Liu Y, Marklund EG (2020). A hydrophobic ratchet entrenches molecular complexes. Nature.

[CR29] Katz LA, Kovner AM (2010). Alternative processing of scrambled genes generates protein diversity in the ciliate *Chilodonella uncinata*. J Exp Zool B.

[CR30] Kimura M (1968). Evolutionary rate at the molecular level. Nature.

[CR31] Kimura M (1979). Model of effectively neutral mutations in which selective constraint is incorporated. Proc Natl Acad Sci USA.

[CR32] Kondrashov FA, Kondrashov AS (2006). Role of selection in fixation of gene duplications. J Theor Biol.

[CR33] Kondrashov FA, Koonin EV (2004). A common framework for understanding the origin of genetic dominance and evolutionary fates of gene duplications. Trends Genet.

[CR34] Landweber LF, Gilbert W (1993). RNA editing as a source of genetic variation. Nature.

[CR35] Lenski RE (2003). The eyes have it. Nature.

[CR36] Levasseur A, Pontarotti P (2011). The role of duplications in the evolution of genomes highlights the need for evolutionary-based approaches in comparative genomics. Biol Direct.

[CR37] Lukeš J, Archibald JM, Keeling PJ (2011). How a neutral evolutionary ratchet can build cellular complexity. IUBMB Life.

[CR38] Lukeš J, Leander BS, Keeling PJ (2009). Cascades of convergent evolution: the corresponding evolutionary histories of euglenozoans and dinoflagellates. Proc Natl Acad Sci USA.

[CR39] Lynch M (2007). The frailty of adaptive hypotheses for the origins of organismal complexity. Proc Natl Acad Sci USA.

[CR40] Lynch M (2018). Phylogenetic divergence of cell biological features. eLife.

[CR41] Lynch M (2013). Evolutionary diversification of the multimeric states of proteins. Proc Natl Acad Sci USA.

[CR42] Lynch M, Blanchard J, Houle D (1999). Perspective: spontaneous deleterious mutation. Evolution.

[CR43] Lynch M, Trickovic B (2020). A theoretical framework for evolutionary cell biology. J Mol Biol.

[CR44] Maurer-Alcalá XX, Nowacki M (2019). Evolutionary origins and impacts of genome architecture in ciliates. Ann N Y Acad Sci.

[CR45] McShea DW, Brandon RN (2010). Biology’s first law: the tendency for diversity and complexity to increase in evolutionary systems.

[CR46] Melnikov S, Manakongtreecheep K, Söll D (2018). Revising the structural diversity of ribosomal proteins across the three domains of life. Mol Biol Evol.

[CR47] Muñoz-Gómez SA, Mejía-Franco FG, Durnin K (2017). The new red algal subphylum proteorhodophytina comprises the largest and most divergent plastid genomes known. Curr Biol.

[CR48] Muñoz-Gómez SA, Snyder SN, Montoya SJ, Wideman JG (2020). Independent accretion of TIM22 complex subunits in the animal and fungal lineages. F1000Research.

[CR49] Nilsen TW (2003). The spliceosome: the most complex macromolecular machine in the cell?. BioEssays News Rev Mol Cell Dev Biol.

[CR50] Noller HF, Hoffarth V, Zimniak L (1992). Unusual resistance of peptidyl transferase to protein extraction procedures. Science.

[CR51] Ohno S (1970). Evolution by gene duplication.

[CR52] Ohta T (1973). Slightly deleterious mutant substitutions in evolution. Nature.

[CR53] Orlenko A, Hermansen RA, Liberles DA (2016). Flux control in glycolysis varies across the tree of life. J Mol Evol.

[CR54] Orlenko A, Teufel AI, Chi PB, Liberles DA (2016). Selection on metabolic pathway function in the presence of mutation-selection-drift balance leads to rate-limiting steps that are not evolutionarily stable. Biol Direct.

[CR55] Osawa S, Jukes TH (1989). Codon reassignment (codon capture) in evolution. J Mol Evol.

[CR56] Papp B, Pál C, Hurst LD (2003). Dosage sensitivity and the evolution of gene families in yeast. Nature.

[CR57] Petrov AS, Wood EC, Bernier CR (2019). Structural patching fosters divergence of mitochondrial ribosomes. Mol Biol Evol.

[CR58] Pillai AS, Chandler SA, Liu Y (2020). Origin of complexity in haemoglobin evolution. Nature.

[CR59] Prescott DM, Greslin AF (1992). Scrambled actin I gene in the micronucleus of *Oxytricha nova*. Dev Genet.

[CR60] Proulx SR (2012). Multiple routes to subfunctionalization and gene duplicate specialization. Genetics.

[CR61] Ramrath DJF, Niemann M, Leibundgut M (2018). Evolutionary shift toward protein-based architecture in trypanosomal mitochondrial ribosomes. Science.

[CR62] Read LK, Lukeš J, Hashimi H (2016). Trypanosome RNA editing: the complexity of getting U in and taking U out. Wiley Interdiscip Rev RNA.

[CR63] Roger AJ, Muñoz-Gómez SA, Kamikawa R (2017). The origin and diversification of mitochondria. Curr Biol.

[CR64] Rzeszutek I, Maurer-Alcalá XX, Nowacki M (2020). Programmed genome rearrangements in ciliates. Cell Mol Life Sci.

[CR65] Sniegowski PD, Gerrish PJ, Lenski RE (1997). Evolution of high mutation rates in experimental populations of *E. coli*. Nature.

[CR66] Sorrells TR, Johnson AD (2015). Making sense of transcription networks. Cell.

[CR67] Speijer D (2006). Is kinetoplastid pan-editing the result of an evolutionary balancing act?. IUBMB Life.

[CR68] Stoltzfus A (1999). On the possibility of constructive neutral evolution. J Mol Evol.

[CR69] Stoltzfus A (2012). Constructive neutral evolution: exploring evolutionary theory’s curious disconnect. Biol Direct.

[CR70] Stuart K (1993). The RNA editing process in *Trypanosoma brucei*. Semin Cell Biol.

[CR71] Tworowski D, Feldman AV, Safro MG (2005). Electrostatic potential of aminoacyl-tRNA synthetase navigates tRNA on its pathway to the binding site. J Mol Biol.

[CR72] Van Leuven JT, Meister RC, Simon C, McCutcheon JP (2014). Sympatric speciation in a bacterial endosymbiont results in two genomes with the functionality of one. Cell.

[CR73] Wedel MJ (2011). A monument of inefficiency: the presumed course of the recurrent laryngeal nerve in sauropod dinosaurs. Acta Palaeontol Pol.

[CR74] Wideman JG, Novick A, Muñoz-Gómez SA, Doolittle WF (2019). Neutral evolution of cellular phenotypes. Curr Opin Genet Dev.

[CR75] Zhang J (2018). Neutral Theory And Phenotypic Evolution. Mol Biol Evol.

[CR76] Zimmer C (2013). The surprising origins of evolutionary complexity. Sci Am.

[CR77] ZuckerkandI E (1997). Neutral and nonneutral mutations: the creative mix—evolution of complexity in gene interaction systems. J Mol Evol.

